# Gut dysbiosis is associated with the reduced exercise capacity of elderly patients with hypertension

**DOI:** 10.1038/s41440-018-0110-9

**Published:** 2018-10-05

**Authors:** Yanbo Yu, Genxiang Mao, Jirong Wang, Liyue Zhu, Xiaoling Lv, Qian Tong, Yefei Fang, Yinxiang Lv, Guofu Wang

**Affiliations:** 1 Wenzhou Medical University, 325035 Wenzhou, Zhejiang China; 2 Department of Geriatrics, Xinchang People’s Hospital, 312500 Shaoxing, Zhejiang China; 3 Department of Geriatrics, Zhejiang Hospital, 310013 Hangzhou, Zhejiang China; 4 Department of Oncology, Xinchang People’s Hospital, 312500 Shaoxing, Zhejiang China

**Keywords:** Gut microbiome, Exercise capacity, 16 s rRNA, Elderly patient, Hypertension

## Abstract

Hypertension is a global health issue, and a reduced exercise capacity is unavoidable for older people. According to recent clinical studies, the intestinal microbiota play a crucial role in the pathogenesis of many human diseases. We investigated whether specific alterations in the gut microbiota contribute to the reduced exercise capacity of elderly patients with hypertension. This study enrolled 56 subjects, and all patients performed a cardiopulmonary exercise test and underwent fecal bacteria sequencing (16 s ribosomal RNA V4 region). According to peak oxygen uptake values, patients were divided into three groups (Weber *A* = 19, Weber *B* = 20, and Weber *C* = 17). The alpha diversity was not significantly different among the three groups. Regarding the beta diversity, Weber A samples were separate from the other two groups in the nonmetric multidimensional scaling ordination plot (ANOSIM pairwise comparisons generated an *R* > 0.5; *p* < 0.05). The abundance of Betaproteobacteria, Burkholderiales, Alcaligenaceae, Faecalibacterium and Ruminococcaceae was diminished in subjects with a reduced exercise capacity (LDA score > 4.0). *Escherichia coli* are a primary producer of trimethylamine and inflammation in the human gut, and the abundance of this bacteria was increased in patients with a reduced exercise capacity (LDA score > 4.0). On the other hand, Lachnospiraceae-*Eubacterium_hallii*_group, Lachnospiraceae-*Lachnoclostridium*, Lachnospiraceae-*Blautia*-*Ruminococcus*_sp__5_1_39BFAA, and Ruminococcaceae-*Faecalibacterium* belong to the order Clostridiales that are likely to produce short-chain fatty acids (LDA score > 4.0), and some of these species were enriched in the Weber B or Weber C group in multiple comparisons. Our data pointed to an altered gut microbiota as a potential contributor to the pathogenesis and progression of the reduced exercise capacity of elderly patients with hypertension.

## Introduction

In recent decades, hypertension has become a major global health burden and one of the most common chronic diseases in the elderly. [1] For elderly people, a reduced exercise capacity is an unavoidable condition. Minimal stressors cause functional impairments in individuals suffering from physical frailty, which is a geriatric syndrome that enhances an individual’s vulnerability. [2] To date, evidence on the validity of interventions to prevent frailty in older people is scarce. [3] Therefore, studies aiming to obtain deeper insights into the pathogenic factors contributing to this condition are urgently needed.

The human gastrointestinal tract is inhabited by a large microbiota, which plays important roles in human health and disease. [4] Recently, accumulating evidence has suggested a link between gut dysbiosis and various disorders, such as hypertension, cardiovascular diseases (CVDs), atherosclerosis, type 2 diabetes and obesity. [5, 6] Many potential mechanisms by which the gut microbiota contributes to diseases have been revealed. For example, metabolic diseases correlate with a chronic state of systemic low-grade inflammation that is related to the clinical disease severity, and the intestinal microbiota is a major source of this pathomechanism. [7, 8] In patients with chronic heart failure characterized by a reduced exercise capacity, the gut overgrowth of pathogenic bacteria such as *Shigella*, *Yersinia*, *Salmonella,* and *Candida* species has been observed. [9] Moreover, trimethylamine (TMA), a microbial metabolite of dietary choline and L-carnitine, is absorbed via the intestinal epithelium and further oxidized to trimethylamine N-oxide (TMAO), which promoted atherosclerosis and aggravate cardiovascular disease in several studies. [10, 11] In contrast, short-chain fatty acids (SCFAs) produced by a number of gut microbiota play a protective role in many chronic diseases. [12, 13] Therefore, changes in the composition and structure of the gut microbiome are pathogenic factors for many diseases. The present study was conducted in elderly patients with hypertension to determine whether changes in the intestinal microbiota are associated with a reduced exercise capacity.

## Methods

### Study population

This study enrolled 56 patients with hypertension from Hangzhou, a large modern city in southern China, who volunteered to participate. Subjects who met the following criteria were included: patients diagnosed with primary hypertension; aged from 65 to 80 years; and lived independently. Exclusion criteria were: any contradictions for cardiopulmonary exercise testing (CPET), uncontrolled hypertension despite the administration of medical therapy, smoking, infection, cancer, stroke, peripheral artery disease, renal failure, water retention, endocrine disorders (e.g., thyroid disease), and inflammatory or malabsorptive intestinal diseases. In addition, the patients had not undergone any laxative, antidiarrheal, steroid, probiotic, and antibiotic treatments within the past 3 months. The participants’ demographic, clinical, and functional characteristics are shown in Table 1. In addition, no statistically significant differences in medication use were observed among groups (Supplementary Table 1).

**Table 1:** Demographic, clinical and functional characteristics of patients

	Weber A (*n* = 19)	Weber B (*n* = 20)	Weber C (*n* = 17)	*P*-value
Age (years)	71.79 ± 1.39	73.5 ± 1.39	75.63 ± 1.10	0.15
Female sex (%)	26.3	40	64.7	0.064
BMI (kg/m²)	23.58 ± 0.56	24.69 ± 0.69	24.80 ± 0.91	0.413
Peak VO_2_/Kg (ml/kg/min)	22.17 ± 0.51* ^†^	18.32 ± 0.25^†^	14.24 ± 0.32	<0.001
VO_2_/Kg at AT (ml/kg/min)	16.82 ± 0.66* ^†^	13.98 ± 0.54^†^	10.71 ± 0.58	<0.001
VTmax (L)	1.53 ± 0.06 ^†^	1.4 ± 0.05^†^	1.09 ± 0.06	<0.001
SBP (mmHg)	152 [128–66]	145.5 [130.25–55.50]	141 [133.25–71.25]	0.38
DBP (mmHg)	80.68 ± 2.47	78.90 ± 2.45	79.50 ± 2.31	0.864
HRmax (bpm)	126.79 ± 3.58	123.7 ± 3.24	116.71 ± 5.40	0.23
BUN (mmol/L)	4.49 [3.85–5.54]	4.76[4.20–5.10]	4.52[4.18–6.20]	0.756
Scr (μmol/L)	70.35 ± 3.26	73.28 ± 3.80	69.15 ± 4.31	0.729
AST (IU/l)	23.10 [19.90–28.30]	25.05 [19.93–27.55]	22.60 [19.05–29.9]	0.955
ALT (IU/l)	17.50 [13.10–26.40]	15.40 [11.75–20.53]	16.90 [11.60–27.00]	0.375
CRP, mg/L nv 0–3 mg/L	0.48 [0.31–83] ^†^	0.81 [0.51–1.96]	1.38 [0.76–3.16]	0.004
LDL-C (mmol/L)	2.34 ± 0.75	2.15 ± 0.83	2.58 ± 0.80	0.929
HDL-C (mmol/L)	1.26 ± 0.21	1.35 ± 0.43	1.30 ± 0.34	0.934
TC (mmol/L)	4.19 ± 0.21	4.06 ± 0.23	4.52 ± 0.22	0.926
TG (mmol/L)	1.19 [0.90–1.59]	1.02[0.83–2.05]	1.50 [0.95–2.06]	0.253
FINS (mg/dL)	67.05 [48.38–80.34]	61.98 [43.94–89.06]	76.76 [38.78–125.05]	0.844
FBG (mmol/L)	5.31 [4.59–6.57]	5.41 [4.89–5.85]	5.37 [5.13–5.88]	0.879
HbA1c (%)	6.20 [5.80–6.80]	6.00 [5.75–6.58]	6.20 [5.85–6.60]	0.777
DM type II (%)	42.1	10	17.6	0.048

*BMI* body mass index, *Peak VO2* peak oxygen uptake, *AT* anaerobic threshold, *VT* tidal volume, *SBP* systolic blood pressure, *DBP* diastolic blood pressure, *HR* heart rate, *BUN* blood urea nitrogen, *Scr* serum creatinine, *AST* aspartate transaminase, *ALT* alanine transaminase, *CRP* C-reactive protein, *LDL-C* low-density lipoprotein cholesterol, *HDL-C* high-density lipoprotein cholesterol, *TC* total cholesterol, *TG* total triglyceride, *FINS* fasting insulin, *FBG* fasting blood glucose, *HbA1c* hemoglobin A1C, *DM type II* diabetes mellitus type 2, *SEM* standard error of mean, *IQR* interquartile range, *Values are %* mean ± SEM or median (IQR)

*, ^†^ *P* < 0.05 compared with the Weber B and Weber C, respectively

This study was approved by the Medical Ethics Committee of Zhejiang Hospital, and the procedures were performed in accordance with the Declaration of Helsinki. All subjects provided written informed consent. The trial was registered in the Chinese Clinical Trial Registry (ChiCTR-IOR-17011799).

### Assessment of clinical parameters

The anthropometric variables were measured, and body mass index (BMI) was calculated according to the following formula: weight/height². Blood variables were determined in fasting venous blood samples. CPET (Cosmed, Rome) was performed according to the standard Bruce protocol to evaluate peak oxygen uptake (peak VO_2_), a measure of exercise capacity.

### Sampling and DNA extraction

Fresh stool samples were collected in standard stool collection tubes one day after enrollment and stored at −80 °C. DNA was extracted from the fecal samples according to the QIAamp Fast DNA Stool Mini Kit (Qiagen, Hilden) operation manual. All DNA samples were frozen at −80 °C until further processing.

### 16 s rRNA PCR and sequencing

Extracted DNA samples were used as template for amplification the bacterial 16 s rRNA gene V4 region using specific primers (515 F 5′-GTGCCAGCMGCCGCGGTAA-3′ and 806 R 5′-GGACTACHVGGGTWTCTAAT-3′) with the barcode. [14] PCR was performed in 30 µl reactions with 15 µl of Phusion® High-Fidelity PCR Master Mix; 10 ng of template DNA, 3 µl (6 µM) of forward and reverse primers, and 2 µl of double-distilled H_2_O. Thermal cycling consisted of initial denaturation at 98 °C for 1 min, followed by 30 cycles of 98 °C for 10 s, 50 °C for 30 s, 72 °C for 30 s and a final extension at 72 °C for 5 min. After quantification and qualification, amplicons were mixed in equidensity ratios. Then, the samples were purified using the Qiagen Gel Extraction Kit (Qiagen, Hilden). Sequencing libraries were produced using a TruSeq® DNA PCR-Free Sample Preparation Kit (Illumina, San Diego) according to the manufacturer’s instructions. Then, the library quality was assessed on the Qubit@ 2.0 Fluorometer (Thermo Scientific) and Agilent Bioanalyzer 2100. Finally, the library was sequenced on the IlluminaHiSeq2500 platform and 250 bp paired-end reads were generated.

### Sequencing analysis

Paired-end reads from the original DNA fragments were merged using FLASH software. Sequences were analyzed using the QIIME software package (Quantitative Insights Into Microbial Ecology), and in-house Perl scripts were used to analyze alpha (within samples) and beta (among samples) diversity. Sequences with ≥97% similarity were assigned to the same operational taxonomic units (OTUs). We chose representative sequences for each OTU and used the RDP classifier to annotate taxonomic information for each representative sequence. Relative abundance was calculated at the phylum and genus levels. We rarified the OTU table and computed three metrics to calculate the alpha diversity: Observed Species, Chao1, and Shannon index. QIIME calculates both weighted and unweighted unifrac values, which are phylogenetic measures of beta diversity. Distance ordination was plotted using the nonmetric multidimensional scaling (NMDS) method. Tests were conducted with linear discriminate analysis (LDA) effect size (LefSe) and ANOSIM.

### Statistics

The Chi-square test was used to analyze enumeration data. Continuous data were compared using ANOVAs, and the LSD-t test was used for multiple comparisons. If numerical data violated the normal distribution and homogeneity of variance, the nonparametric Kruskal-Wallis H test was used to compare data among groups and a Dunn-Bonferroni test was used for post hoc comparisons. Spearman’s rank correlation coefficient was calculated to estimate the linear correlations between variables. All statistical analyses were completed using the SPSS 19.0 software. Statistical analyses of sequencing data were performed using the R package (version 2.15.3) and tools such as Chao 1, Simpson, Shannon, non-metric multidimensional scaling (NMDS), ANOSIM, and LefSe. *P* < 0.05 was considered statistically significant.

## Results

### Demographic, clinical, and functional profiles of study participants

Based on the peak VO_2_ values obtained during CPET, patients were divided into three functional classes according to Weber’s classification system: 19 patients were in class A (normal exercise capacity), 20 in class B and 17 in class C (reduced exercise capacity). Significantly higher levels of the plasma inflammatory biomarker CRP were observed in the Weber A group compared to the Weber C group (*p* = 0.004). Although the increase in CRP levels in Weber B and Weber C groups was not clinically significant, we detected a trend towards systemic low-grade inflammation in the patients with hypertension who presented a reduced exercise capacity. Peak VO_2_/kg and VO_2_/kg at the anaerobic threshold (AT) were both significantly different between groups according to the multiple comparisons test (*p* < 0.01). The VTmax in the Weber C group was significantly lower than the Weber A (*p* < 0.001) and Weber B groups (*p* = 0.001), respectively. Notably, the ratio of DM type II was statistically significantly different in the global comparison, but no difference was observed in the multiple comparisons test (Table 1).

### Intestinal microbiome

**Fig. 1:**
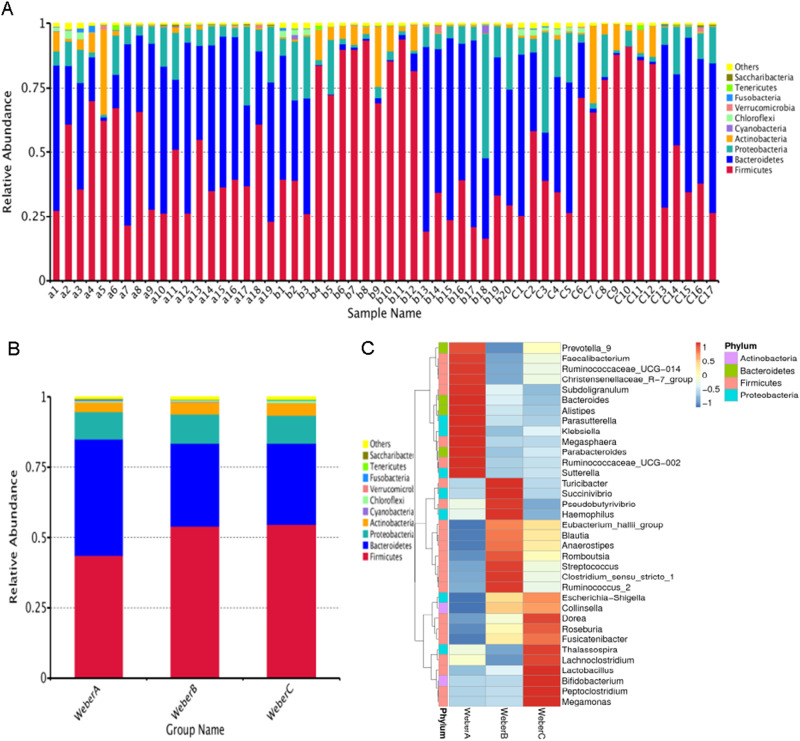
**a** Relative abundances of the top 10 phyla by composition in individual samples. **b** Relative abundances of the top 10 phyla by composition in the three groups. **c** The abundances of the top 35 bacteria at the genus level in each group are shown. The heat map is color-coded based on row Z-scores. The groups with the highest and lowest bacterial abundances are shown in red and blue, respectively

**Fig. 2:**
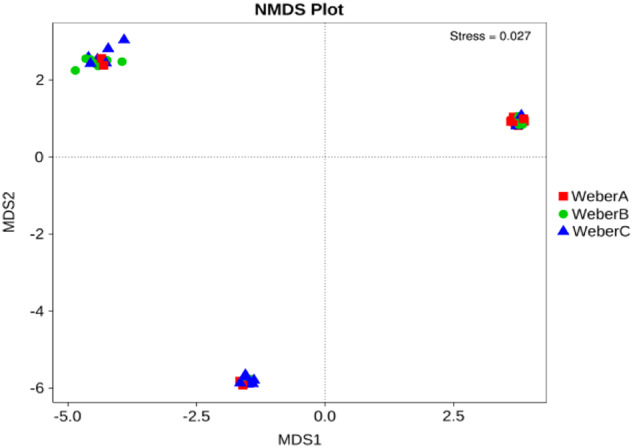
Nonmetric multidimensional scaling (NMDS) ordination plot of the Weber A, Weber B, and Weber C samples. Ordination was based on Bray-Curtis dissimilarity calculated with OTU-level data. Each point represents one sample; the closer the points are located to one another, the more similar the microbiome compositions of the samples

The composition of the gut microbiota of all patients was mainly dominated by the 4 phyla: Firmicutes, Bacteroidetes, Proteobacteria, and Actinobacteria (Fig. 1a, b). The ratio of members of the phylum Firmicutes to Bacteroidetes (F/B ratio) is considered a potential biomarker of pathological conditions. [15] Although patients in the Weber A group had a lower F/B ratio than the other two groups, the difference was not statistically significant (Supplementary Table 2). Next, an overview of the abundances of the top 35 genera in the three groups is presented (Fig. 1c). However, we did not detect significant differences among three groups in the relative abundances of the major phyla or in alpha diversity measures (Chao 1, Simpson, Shannon; Kruskal–Wallis *H* test, *p* > 0.05). Regarding beta diversity, which represents interindividual variances, the NMDS plots based on OTU distribution showed partial overlapping distributions among each group (Fig. 2), and Weber A samples were still separate from the other groups (ANOSIM pairwise comparisons generated an R > 0.5, *p* < 0.05).

**Fig. 3:**
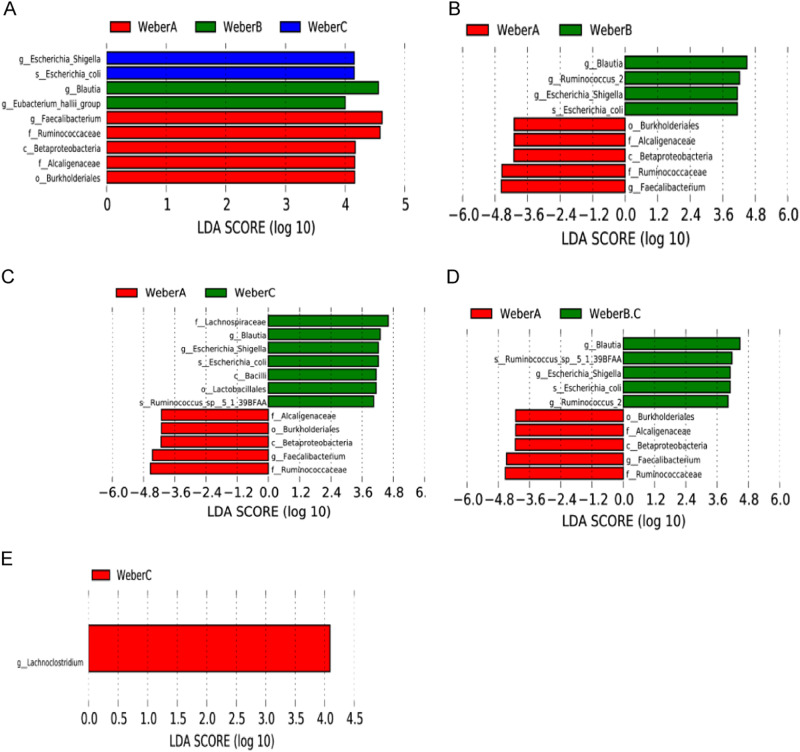
A LefSe analysis along with linear discriminate analysis (LDA) was applied to identify indicator bacterial in the groups. **a** Weber A vs. Weber B vs. Weber C, (**b**) Weber A vs. Weber B, (**c**) Weber A vs. Weber C, (**d**) Weber A vs. Weber B + C and (**e**) Weber B vs. Weber C

The results of the LefSe analysis (LDA score > 4.0, *p* < 0.05) performed on the relative abundance data for the microbiota are presented in Fig. 3. In terms of the overall situation (Fig. 3A), the abundance of the class Betaproteobacteria was prominently increased in the Weber A group, including the order Burkholderiales and the family Alcaligenaceae; the family Ruminococcaceae and the genus *Faecalibacterium* were also enriched in the Weber A group compared to the other two groups. The genus *Escherichia_Shigella* and the species *Escherichia coli* were significantly increased in the Weber C group, and the genus *Blautia* and *Eubacterium hallii* were enriched in the Weber B group. In pairwise comparisons, the gut microbes mentioned above that were enriched in Weber A were decreased in the Weber B, Weber C and Weber B + C (Weber B. C) groups (Fig. 3b–d). The abundances of the genera *Blautia*, *Ruminococcus*_2, *Escherichia_Shigella* and the species *Escherichia coli* (*E. coli*) were significantly increased in the Weber B group compared to the Weber A group (Fig. 3b). The relative abundances of the class Bacilli, the order Lactobacillales, the family Lachnospiraceae, the genera *Blautia* and *Escherichia_Shigella*, the species *E. coli* and *Ruminococcus*_sp__5_1_39BFAA were enriched in the Weber C group compared to the Weber A group (Fig. 3c), The abundances of the genera *Blautia*, *Escherichia_Shigella*, *Ruminococcus*_2, the species *E. coli* and *Ruminococcus*_sp__5_1_39BFAA were significantly increased in the Weber B. C group (Fig. 3d). Consistent with the alpha and beta diversity values, little difference was observed between the Weber B and Weber C groups (Fig. 3e).

### A reduced exercise capacity negatively associates with the core gut microbiota

We next determined the correlations between the core gut microbiota and clinical parameters of patients (Table 2). The bacterial counts of Lactobacillales, *Eubacterium_hallii*_group and *Blautia* were positively correlated with CRP levels (*r* = 0.385, *p* = 0.003; *r* = 0.296, *p* = 0.027 and *r* = 0.268, *p* = 0.046; respectively), while Alcaligenaceae was negatively correlated with CRP levels (*r* = −0.294, *p* = 0.028). Bacterial counts of the Lactobacillales, *Blautia*, *Ruminococcu*s_sp._5_1_39BFAA and *E. coli* were negatively correlated with the peak VO_2_/kg (*r* = −0.284, *p* = 0.034; *r* = −0.290, *p* = 0.030; *r* = −0.273, *p* = 0.042 and *r* = −0.355, *p* = 0.007; respectively), while Alcaligenaceae was positively correlated with peak VO_2_/kg levels (*r* = 0.318, *p* = 0.017). Meanwhile, the *E. coli* count negatively correlated with VO_2_/kg at AT (*r* = −0.364, *p* = 0.006).

**Table 2:** Correlation analysis of clinical values and the relative abundance of core gut microbiota

		o_Lactobacillales	f_Alcaligenaceae	g_Eubacterium_hallii_group	g_Blautia	g_Faecalibacterium	g_Lachnoclostridium	g_Ruminococcus_2	s_Ruminococcus_sp._5_1_39BFAA	s_Escherichia_coli
FBG	*r*	0.553**	−0.149	0.260	0.249	−0.067	0.119	0.089	0.249	0.110
	*p*	0.000	0.273	0.053	0.064	0.622	0.384	0.515	0.064	0.420
BUN	*r*	0.109	−0.225	0.281*	0.297*	−0.309*	−0.027	0.277*	0.312*	0.239
	*p*	0.426	0.095	0.036	0.026	0.021	0.842	0.039	0.019	0.076
Scr	*r*	−0.011	−0.111	0.121	0.085	−0.137	0.030	0.320*	0.106	0.061
	*p*	0.934	0.415	0.373	0.533	0.313	0.825	0.016	0.436	0.653
CRP	*r*	0.385**	−0.294*	0.296*	0.268*	−0.126	−0.048	0.018	0.245	0.148
	*p*	0.003	0.028	0.027	0.046	0.355	0.726	0.895	0.068	0.276
HbA1c	*r*	0.325*	−0.121	0.086	0.101	0.015	0.047	−0.115	0.103	-0.025
	*p*	0.015	0.373	0.526	0.457	0.910	0.729	0.399	0.448	0.855
Peak VO2/Kg	*r*	−0.284*	0.318*	−0.263	−0.290*	0.159	−0.143	−0.182	-0.273*	-0.355**
	*p*	0.034	0.017	0.050	0.030	0.241	0.293	0.181	0.042	0.007
VO2/Kg at AT	*r*	0.004	0.114	−0.123	−0.112	0.067	−0.050	−0.023	-0.068	-0.364**
	*p*	0.975	0.404	0.366	0.412	0.623	0.714	0.868	0.621	0.006
BMI	*r*	0.371**	−0.249	0.273*	0.242	−0.099	0.065	0.212	0.273*	0.123
	*p*	0.005	0.066	0.044	0.074	0.474	0.637	0.120	0.044	0.373
SBP	*r*	−0.128	−0.073	−0.023	−0.003	0.136	0.158	−0.010	0.009	-0.253
	*p*	0.353	0.598	0.869	0.982	0.322	0.248	0.942	0.947	0.063
DBP	*r*	−0.050	0.048	−0.130	−0.051	0.014	0.038	−0.132	-0.035	-0.080
	*p*	0.718	0.726	0.345	0.713	0.922	0.784	0.338	0.802	0.562
Age	*r*	0.180	−0.119	0.085	0.070	0.001	0.268*	0.221	0.110	0.064
	*p*	0.185	0.382	0.533	0.608	0.992	0.046	0.101	0.418	0.640

*Note*: Spearman rank correlation was used to evaluate statistical importance: *r*: correlation coefficient

*FBG* fasting blood glucose, *BUN* blood urea nitrogen, *Scr* serum creatinine, *CRP* C-reactive protein, *HbA1c* hemoglobin A1C, *Peak VO2* peak oxygen uptake, *AT* anaerobic threshold, *BMI* body mass index, *SBP* systolic blood pressure, *DBP* diastolic blood pressure

*p < 0.05, **p < 0.01

## Discussion

Based on accumulating evidence obtained from recent studies, gut dysbiosis and induces a chronic state of systemic low-grade inflammation that plays a crucial role in the pathophysiology of CVDs, including hypertension. [7, 16] Hypertension is a chronic disease that is considered a main risk factor for the development and progression of CVDs and displays a low control rate and high prevalence in several populations, particularly elderly people in developing countries. [17] On the other hand, physical exercise is possibly the most promising nonpharmacological approach to prevent and treat hypertension, and the optimal levels of exercise capacity are associated with lower all-cause and cardiovascular mortality and morbidity. [18, 19] Interestingly, robust associations between the effects of age and frailty on both the composition and function of the gut microbiome were identified. [20] Therefore, we investigated the relationship between the gut microbiota and exercise capacity in the elderly patients with hypertension.

To our knowledge, this study is the first systematic analysis of the correlation between the intestinal microbiota and reduced exercise capacity in elderly patients with hypertension using high-throughput sequencing of bacterial 16 s rRNA gene sequences. Because all samples were obtained from elderly patients with hypertension, a difference in the alpha diversity was not identified in this analysis. A reduced exercise capacity is one of the most common clinical symptoms of stable heart failure and many other chronic diseases. Recently, alterations in the composition and metabolism of the intestinal microbiota have been identified as pathogenic factors contributing to the development of atherosclerosis and CVD. [16, 21] Notably, TMAO, the hepatic oxidation product of the gut microbial metabolite TMA, has attracted increasing attention as a potential promoter of atherosclerosis. [22] Two independent cohort studies suggested that plasma concentrations of TMAO predict an enhanced risk of major adverse cardiac incidents. [23] Moreover, *E. coli* is a primary producer of TMA and inflammation in humans. [22] Interestingly, the abundances of *Escherichia_Shigella* and *E. coli* were higher in patients with a reduced exercise capacity than in patients with a normal exercise capacity and were negatively correlated with exercise capacity. Additionally, CRP levels were increased in the Weber C group compared to the Weber A group, although a clinical implication was not determined. This finding is consistent with a previous report showing a correlation between increased CRP levels and the intestinal abundance of pathological *Shigella* species in patients with moderate-to-severe heart failure. [9] Therefore, we concluded that *E. coli* may play an important role in decreasing the exercise capacity of elderly patients with hypertension.

SCFAs are produced by specific gut microbes through the fermentation of soluble fiber and are predicted to have anti-inflammatory properties. [24, 25] The predominant intestinal bacteria that produce SCFAs are classified as Eubacterium (cluster XIVa) and Ruminococcaceae (cluster IV) in the order Clostridiales, and class Clostridia. [25] Additionally, Lachnospiraceae possess rich carbohydrate-active enzyme (CAZyme) repertoires that utilize complex polysaccharides to form the major energy source for several Clostridium XIVa strains and colon-resident bacteria. [22, 26] In our study, Lachnospiraceae-*Eubacterium_hallii*_group, Lachnospiraceae-*Lachnoclostridium*, Lachnospiraceae-*Blautia*-*Ruminococcus*_sp__5_1_39BFAA, and Ruminococcaceae-*Faecalibacterium* belong to the order Clostridiales; some of these species were enriched in the Weber B or Weber C group in multiple comparisons tests. However, the bacterial counts of Lactobacillales and *Blautia* were positively correlated with CRP levels and were negatively associated with peak VO_2_/kg. Hence, we deduced that the gut may employ a compensatory mechanism to alleviate the actions of pathogenic bacteria. Moreover, the abundances of Betaproteobacteria, Burkholderiales, and Alcaligenaceae were diminished in subjects with a reduced exercise capacity and were positively correlated with peak VO_2_/kg and were negatively correlated with CRP levels. This study is the first to focus on correlations between the alterations in the gut microbiota with reduced exercise capacity in elderly individuals with hypertension, and it provided a new concept of this condition for further exploration. However, the present study has several limitations. First, we did not examine the serum concentrations of TMAO and SCFAs; therefore, we were unable to directly determine the relationship between the intestinal microbiota and TMAO or SCFAs. Second, our results were based on relatively small sample sizes, and further large-scale studies are needed to prove our findings. Finally, comprehensive metagenomic and metabolomic analyses are needed in the future to perform a more in-depth assessment of the gut microbiota composition and metabolism in elderly subjects.

In conclusion, a striking negative correlation between a reduced exercise capacity and specific taxonomic abundances. Intestinal dysbiosis plays a potential pathogenic role in reducing the exercise capacity of elderly patients with hypertension. Further research is needed in this area to provide additional supporting evidence for the development of a novel specific therapy or strategy to alleviate this condition.

## Electronic supplementary material

Supplementary Table 1

Supplementary Table 2
