# Supplementary material for: Gut dysbiosis is associated with the reduced exercise capacity of elderly patients with hypertension
Source: Hypertens Res. 2018 Oct 5;41(12):1036–44. doi: 10.1038/s41440-018-0110-9 (PMC8076014; doi:10.1038/s41440-018-0110-9)
Supplement: Supplementary file 1 — Supplementary Table 1 [file 41440_2018_110_MOESM1_ESM.doc]

|  | Weber A (n=19) | Weber B (n=20) | Weber C (n=17) | P-value |
| --- | --- | --- | --- | --- |
| ARB or ACEI | 47.2% | 55.0% | 52.9% | 0.887 |
| CCB | 61.9% | 52.6% | 52.9% | 0.845 |
| Beta-blocker | 33.3% | 35.0% | 17.9% | 0.455 |
| Diuretic | 5.3% | 20.0% | 23.5% | 0.276 |
| Aspirin | 31.6% | 35.0% | 17.6% | 0.476 |
| Statin | 52.6% | 25.0% | 17.6% | 0.056 |
| Metformin | 15.8% | 10.0% | 11.8% | 0.856 |
| Acarbose | 21.1% | 0 | 5.9% | 0.061 |

Supplementary Table 1 Medications of the patients. Abbreviations: ARB, angiotensin receptor blockers; ACEI, angiotensin-

converting enzyme inhibitors; CCB, calcium channel blockers. Values are %.
