# Supplementary material for: Gut dysbiosis is associated with the reduced exercise capacity of elderly patients with hypertension
Source: Hypertens Res. 2018 Oct 5;41(12):1036–44. doi: 10.1038/s41440-018-0110-9 (PMC8076014; doi:10.1038/s41440-018-0110-9)
Supplement: Supplementary file 2 — Supplementary Table 2 [file 41440_2018_110_MOESM2_ESM.doc]

|  | WeberA | WeberB | WeberB | P value |
| --- | --- | --- | --- | --- |
| F/B ratio | 0.86[0.46-2.20] | 1.04[0.60-99.75] | 1.92[0.55-75.58] | 0.328 |

Supplementary Table 2 The Firmcutes/Bacteroidetes ratio (F/B ratio) was calculated as a biomarker of gut dysbiosis. Values are median (IQR).
